# A Proposed Conceptual Sport Nutrition Approach for Athlete Development and Assessment: The Athlete Nutrition Development Approach

**DOI:** 10.1186/s40798-022-00532-w

**Published:** 2022-12-08

**Authors:** Kevin Iwasa-Madge, Erik Sesbreno

**Affiliations:** 1 Canadian Sport Institute Ontario, Toronto, ON Canada; 2 Institut National du Sport du Quebec, Montreal, QC Canada; 3 McGill University, Montreal, QC Canada; 4 French-Speaking Research Network for Athlete Health Protection and Performance (ReFORM), Montreal, QC Canada

**Keywords:** COM-B, Eating behaviours, Food choices, Dietary intake

## Abstract

Appropriate dietary intake can improve athletes’ health and sport performance and is a direct result of eating behaviours. Therefore, assessing and shaping athletes’ eating behaviours and dietary intake is critical to the provision of sport nutrition services. As such, nutrition practitioners must also consider the determinants of eating behaviours. However, dietary intake, eating behaviours, and its determinants are inconsistently defined in the literature, requiring nutrition practitioners to navigate a complicated landscape of concepts and terminology. This is further complicated by limitations in practically measuring and influencing eating behaviours and dietary intake. The proposed Athlete Nutrition Development Approach was developed to aid practitioners in servicing decisions through the athlete development process, through a three-tiered approach to sport nutrition service delivery. Tier 1 addresses the determinants of eating behaviours, Tier 2 directly addresses eating behaviours and dietary intake, and Tier 3 addresses the consequences of dietary intake in relation to health and sport performance. Each tier includes tools for assessment and development.

## Key Points

1. There are many determinants of an athlete’s eating behaviours.
2. Dietary intake is a result of eating behaviours.
3. Appropriate dietary intake can improve an athlete’s health and performance.

## Introduction

Dietary intake has a profound influence on athlete health and sport performance [1]. It is therefore important to account for eating behaviours, given their direct influence on dietary intake [2, 3]. In the most general sense, behaviour has been described as actions and its determinants and consequences [4]. However, eating behaviours, dietary intake, determinants, and consequences are vaguely or inconsistently defined [3, 5]. Some authors have differentiated between behaviours that precede food entering the mouth (food choices), the act of eating (eating behaviours), with eating habits being a subset of eating behaviours, and the results of eating behaviours (dietary intake) [2, 3]. The athlete-centred literature more specifically differentiates habits from behaviours as being regularly repeated behaviours to reduce the need for conscious decision-making [6]. However, older literature describes this combination of conscious and subconscious decision-making as the food choice process [7], while other authors use the term eating routines [8]. Authors have also used the term eating patterns to describe food choices, and the frequency of meals and snacks, which results in nutrient intake (defined similarly to dietary intake) [9]. Additionally, concepts such as nutrition literacy and food literacy use ranging definitions from general health knowledge, to specific skills and competencies required to interact within a food system [5]. Nutrition practitioners working in the field are navigating a complicated landscape of concepts and terminology, compounded with limitations in practically measuring and influencing nutrition-related factors [10, 11].

**Fig. 1:**
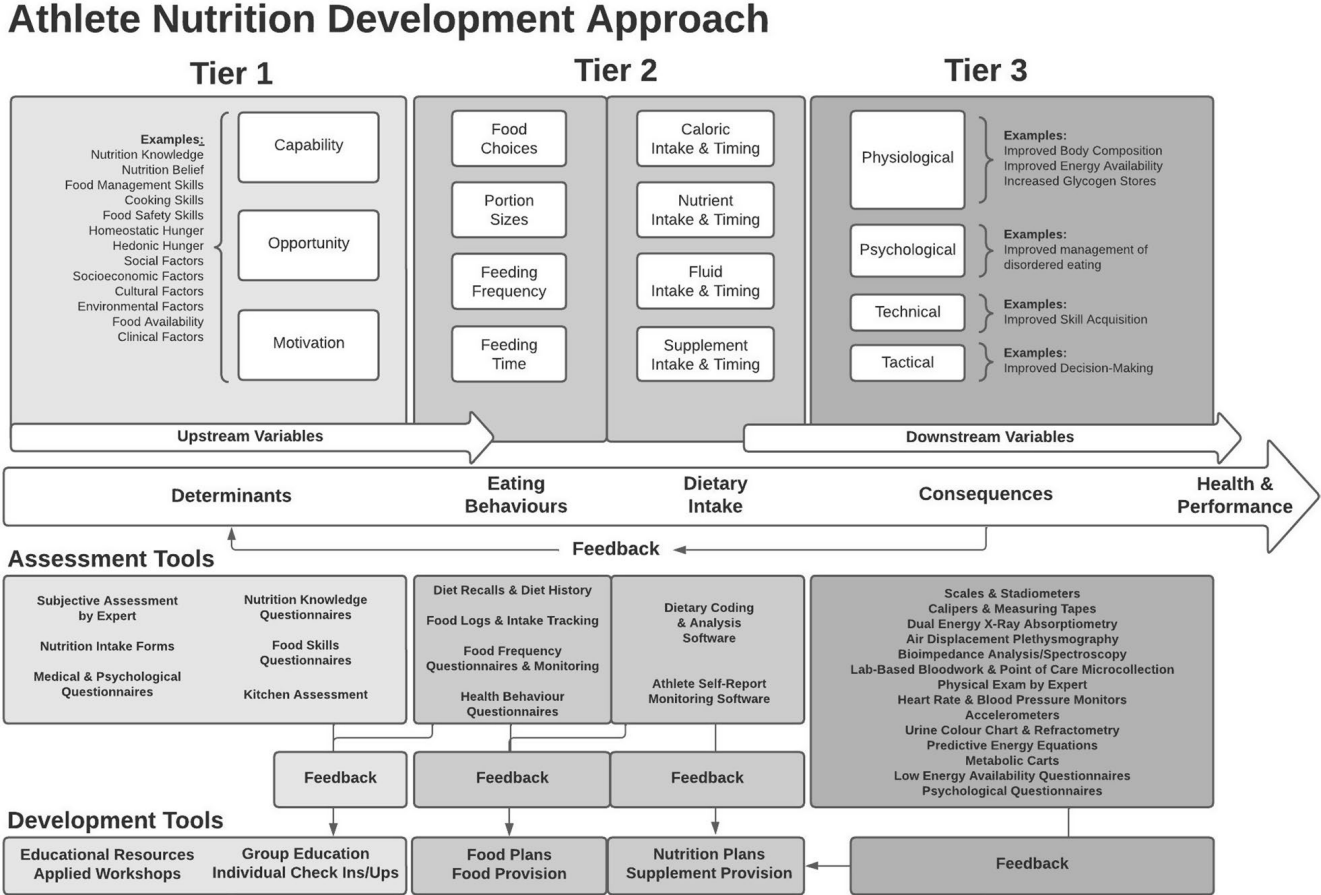
The Athlete Nutrition Development Approach outlining a three-tiered approach that addresses the upstream determinants of eating behaviours (Tier 1), eating behaviours and dietary intake (Tier 2), and the downstream consequences of dietary intake (Tier 3), with the goal of improving athlete health and performance. Each tier includes an overview of the concepts and suggested tools for assessment and development. Figure created using Lucidchart

Unfortunately, there is currently no single agreed-upon approach to improve eating behaviours and dietary intake in athletes. Recent literature outlines nutrition needs for youth athlete development, suggesting various shifts in focus through the athlete development process (e.g. removing body composition assessments, discouraging supplement use, and promoting eating behaviours and dietary intake that support age-appropriate development) [12], but does not provide an operational approach to manage this outcome. Interestingly, a new conceptual framework layers sport nutrition services onto stages of development and skill level [13], but the scope of focus is limited to the assessment of body composition. The Determinants of Nutrition and Eating (DONE) framework and taxonomy uses tiers, including factors that precede eating, the actions of eating, and the results of eating [2, 3], but is not viewed through a sport nutrition lens, nor does it guide servicing decisions. Given the various limitations of each model/framework, the purpose of this paper is explore a conceptual sport nutrition approach that aids practitioners in nutrition-related servicing decisions through various athlete development processes based on an understanding of sport performance, eating behaviours and dietary intake, and their determinants and consequences. This paper proposes a novel Athlete Nutrition Development Approach to establish a tiered approach to sport nutrition services (Fig. 1). Tier 1 focuses on the independent, upstream determinants of eating behaviours; Tier 2 focuses directly on eating behaviours and dietary intake; and Tier 3 focuses on the dependent, downstream consequences of dietary intake. Each tier includes a description of the concepts and is comprised of two sections. The first sections of the approach utilize tools to assess and measure eating behaviours, dietary intake, and their determinants and consequences, while the second section proposes tools to develop and shape eating behaviours, dietary intake, and their determinants and consequences in athletes. Together, these sections form a ‘toolkit’ to guide practitioners in providing sport nutrition services to athletes.

## Components

### Tier 1: Determinants of Eating Behaviours

Eating behaviours are influenced by a complex set of factors, including both modifiable and non-modifiable variables. In this paper, these factors will be referred to as the determinants of eating behaviours and will be described using the Capability, Opportunity, Motivation-Behaviour (COM-B) system [14], given its use within sport nutrition literature to date [15, 16]. Capability is defined as the capacity to engage in a behaviour, requiring knowledge and skill [14]. Opportunity can be described as the external, contextual factors that make a behaviour possible [14]. Lastly, motivation is the brain processes that direct behaviour (both emotional and analytical) [14]. Together, these components are interrelated and create behaviours. It is beyond the scope of this paper to provide an exhaustive list of the determinants of eating behaviours. Rather, some key determinants are described below using the COM-B system. Additionally, this tier describes tools to measure and shape the determinants within these three components to understand and improve eating behaviours.

#### Key Determinants

Nutrition knowledge and beliefs are primary determinants and can be defined as an awareness and ability to apply nutrition information when choosing foods [6]. Importantly, athletes identify a lack of knowledge as a barrier to appropriate dietary intake [17]. Fortunately, nutrition knowledge is modifiable [18], and evidence suggests that athletes may benefit from sport nutrition education through increases in nutrition knowledge, improved eating habits, changes in body composition, and improved physical performance [11]. It is noteworthy that nutrition knowledge and beliefs also serve as a lens through which athletes can interpret, both correctly and incorrectly, the impact of other determinants of eating behaviours (described below), and the actions and consequences of eating and resulting dietary intake, creating an iterative process where the downstream factors described later in this paper can indirectly serve as determinants of eating behaviours [19]. Nutrition knowledge has been considered a ‘Capability’ component of the COM-B system in previous studies [15]. However, beliefs are considered ‘Motivation’ [15]. Food skills such as food management skills, cooking skills, and food safety skills also affect eating behaviours [5] and are considered a ‘Capability’ [15].

Knowledge, skills, and belief alone cannot fully explain eating behaviours. Other modifiable and non-modifiable factors exist and are often complex. Homeostatic and hedonic hunger influence eating behaviours. While an awareness of how hunger can influence eating behaviours may allow for the interpretation of hunger cues to better meet physiological and psychological needs (coded as ‘Capability’ as part of attention and decision processes) [15], hunger itself is independent of knowledge and skill when upstream of eating behaviours and could be viewed as a contextual factor and coded as ‘Opportunity’. Other external ‘Opportunity’ factors that can create barriers and influence eating behaviours can occur on social, socio-economic, cultural, and environmental dynamics [6, 15, 17]. Food availability is complex as it can be influenced by awareness and management skills (‘Capability’) and belief in capability and/or consequences (‘Motivation), but it is also largely influenced by an ‘Opportunity’ factor in many circumstances (i.e. if food is available, it is easier to eat).

Components of the COM-B system are interrelated and form complex interactions that determine eating behaviours. Of particular concern are clinical circumstances such as disordered eating in athlete populations [20] and gastrointestinal disorders [1], which have complex aetiologies that influence eating behaviours. Table 1 contains a list of possible determinants of eating behaviours, and how they are coded within the COM-B system.

**Table 1:** Overview of common determinants of eating behaviours utilizing the Capability, Opportunity, Motivation-Behaviour system, with descriptions and examples

Coding	Determinant	Description	Example
Capability	Nutrition knowledge	Nutrition knowledge is the awareness and ability to apply nutrition information when choosing foods [6]	Knowledge of nutrient and energy content of food Knowledge of nutrient and energy requirements Dieting due to perceived effects on performance and/or body composition
Motivation	Nutrition beliefs	Nutrition beliefs are related to perceived abilities and consequences [15]	Willingness to spend time and effort to prepare food Belief that eating a certain food will improve performance Batch cooking ahead of time
Capability	Cooking, food management, and food safety skills	The ability to access, select, purchase, prepare, and preserve food [5]	Ability to efficiently navigate a grocery store Ability to batch cook Ability to cook food to proper internal temperature Ability to store food safely
Capability and opportunity	Homeostatic hunger	Homeostatic hunger is a complex physiological feedback process that signals the need for food [57]	Energy balance Energy density of food consumed Volume of food consumed Macronutrient profile of food consumed
Capability and opportunity	Hedonic hunger	Hedonic hunger links food with pleasure while interacting with the homeostatic hunger system [57, 58]	Taste and preferences Aesthetic presentation of food
Opportunity	Social, socio-economic, cultural, and environmental factors	Social, socio-economic, cultural, and environmental factors are extrinsic factors altering food availability and autonomy	Financial constraints Customs and traditions Exposure to marketing Religious food restrictions Ethical food restrictions Access to cooking facilities and equipment Access to safe food storage
Capability, opportunity, and motivation	Food availability	Food availability is the access to foods in sufficient quantities at appropriate times	Access to protein-containing foods immediately following training Access to carbohydrate-containing foods in appropriate quantities
Capability, opportunity, and motivation	Clinical factors	Clinical factors are intrinsic factors that can influence nutrient and energy requirements, or add barriers to eating behaviours	Eating Disorders/Disordered Eating Metabolic conditions Allergies and Intolerances Gastrointestinal issues

#### Tier 1: Assessment Tools

Determinants of eating behaviours can be difficult to assess. Practitioners such as Registered Dietitians (RDs) have been trained to subjectively and qualitatively assess these determinants. The Nutrition Care Process and Model (NCPM) is commonly used among RDs and involves four major steps, including nutrition assessment (step 1); nutrition diagnosis (step 2); nutrition intervention (step 3); and nutrition monitoring (step 4) [21]. Step 1 and step 4 provide a standardized approach to assessment and reassessment [21], including the assessment/reassessment of these determinants. Steps 2 and 3 will be described in later sections of this paper. Nutrition assessment can be categorized in an A-E framework, including anthropometric (A), biochemical (B), clinical (C), dietary (D), and environmental (E) assessments [22], with clinical and environmental assessments regarding specific determinants of eating behaviours. Anthropometric, biochemical, and dietary assessment will be described in later sections of this paper. Documentation can occur in a number of ways, but often utilizes a pre-determined structure such as a Subjective, Objective, Assessment, Plan note [23]. An approach such as the NCPM or A-E framework can be highly specific and add a richness to the assessment process, but the individualized and qualitative nature can be time-consuming. Alternatively, subjective components can be quantified through the use of rating or frequency scales, such as components of the Athlete Food Choice Questionnaire [19] or Three-Factor Eating Questionnaire [24], and used within or in addition to subjective nutrition intake forms for quick and widespread distribution.

Nutrition knowledge is one of the main modifiable determinants of eating behaviours [18]. As such, the development of nutrition knowledge, and therefore assessment of nutrition knowledge, is a critical component of early nutrition development. Practitioners will often subjectively and qualitatively assess nutrition knowledge along with other determinants as described earlier in this paper. However, validated sport nutrition knowledge questionnaires exist [25, 26], allowing for a more thorough and quantified assessment of nutrition knowledge. Similarly, validated food skills questionnaires exist [27], providing an opportunity to quantify a different set of determinants, although a subjective assessment by an expert in a kitchen may also be valuable.

Although psychological factors can be dependent on dietary intake, this relationship is often bidirectional with mental health being a determining factor in eating behaviours and resulting dietary intake [28, 29]. Evidence suggests that female athletes may be especially prone to disordered eating and would benefit from screening [30], although disordered eating does also occur in males [29]. Therefore, it is important to assess mental health at early stages of development, and not just as a psychological consequence of dietary intake. Tools, such as the Eating Disorder Examination 17.0, which is considered the gold standard [20, 31], is an option, but the Eating Disorder Examination Questionnaire 6.0, Athlete Milieu Direct Questionnaire Version 2, Brief Eating Disorder in Athletes Questionnaire Version 2, and the Eating Disorder Screen for Athletes, may be more practical options to implement [20, 32, 33]. Interdisciplinary support may be required for assessment in this area.

#### Tier 1: Development Tools

Nutrition counselling and consultations are commonly used in sport nutrition [34]. The NCPM is a model to guide practitioners through standardized nutrition service provision [21]. Nutrition assessment (step 1) and nutrition monitoring (step 4) are two steps that have been discussed earlier. The nutrition intervention (step 3) step involves formulating and delivering a plan of action to address identified problems [21]. When related to the determinants of eating behaviours, this may require: increasing nutrition knowledge; increasing cooking, food safety, and food management skills; improving awareness of homeostatic and hedonic hunger cues; improving awareness of clinical, social, socio-economic, cultural, and environmental barriers; and improving motivation. In addition to direct delivery of information, the use of techniques such as motivational interviewing [35] and intuitive eating [36]; and theoretical approaches to enhancing intrinsic motivation such as self-determination theory [37], and the behaviour change wheel [14], can help athletes develop, although research in athlete populations is lacking. Delivery of these services can occur in both formal appointments with athletes and practitioners (check-ups) and informal communication in the daily training environments (check-ins).

Other types of development tools that work at a group level include education in the form of: presentations [38]; resources such as ‘Athlete Plates’ [39] and infographics [40]; and applied workshops to provide opportunities to practice skills [38]. Resources can be distributed directly to athletes live or virtually, or modification of the physical environment such as posting resources in daily training environments can provide athletes with constant, passive exposure to desired information. The benefits of these services are twofold: they provide athletes with information which aids in development; and they create a positive nutrition culture within and around the athletes. Early adopting athletes can serve as champions [41], helping positive nutrition culture spread within a group of athletes. Additionally, parents, coaches, and friends can all influence nutrition culture [6], and so in some cases, it may be advantageous to provide educational services to an athlete’s entourage to influence the nutrition culture around the athletes.

### Tier 2: Eating Behaviours and Dietary Intake

Behaviours are not consistently defined in the literature, with some definitions focussing on behaviours solely as actions or acts [3], while other definitions include the determinants, correlates, and consequences of actions [4]. Given the focus on determinants and consequences in other areas of this approach, Tier 2 will emphasize eating behaviours as the *actions* related to eating and define dietary intake as the results of eating behaviours. Eating behaviours include food choices, portion sizes, feeding frequency, and feeding time and result in dietary intake: timing and intake of calories, nutrients, fluids, and supplements. Some literature works describe eating habits as a factor interrelated with eating behaviours [3, 6]. Specifically, Birkenhead and Slater [6] describe habits as behaviours that are regularly repeated to reduce the need for conscious decision-making. However, it can be argued that eating habits (and any other synonyms used within the literature) can still be characterized and described through: the actions of food choices, portion sizes, feeding frequency, and feeding time; and the determinants of these actions. Therefore, it is acknowledged that eating habits exist, but will not be a term used in this approach as they are not mutually exclusive of food choices, portion sizes, feeding frequency, and feeding time, and the determinants of these actions. This tier revolves around measuring and influencing eating behaviours and resulting dietary intake to optimize adaptation to training and readiness to perform.

#### Tier 2: Assessment Tools

Commonly, practitioners look to assess both eating behaviours and dietary intake. However, eating behaviours are transient and difficult to assess. Furthermore, to assess dietary intake, eating behaviours must be coded and analysed using software [42]. This process takes time, making the assessment of dietary intake more time-consuming than the assessment of eating behaviours [22, 43]. Error introduced at the dietary intake level through the coding and analysis process is also a concern [44]. This section describes the types of eating behaviour and dietary intake assessment tools available to practitioners.

Possibly the most salient option is the observation of eating behaviours. This provides an objective look at eating behaviours and removes any reporting error introduced by athlete self-monitoring. Observation can be blinded or non-blinded, depending on the circumstances. In non-blinded situations where athletes are aware they are being observed, desirability bias may play a factor. However, blinded observation is not always possible or ethical. Alternatively, prospective assessment of both eating behaviours and dietary intake can be accomplished for a period of time through the use of self-report food logs or intake tracking software [22]. While this approach has the advantage of gathering very detailed eating behaviour data [22], these tools are greatly limited by the athlete and practitioner burden, making them unrealistic to complete on a daily basis as well as introducing reporting errors [10, 22]. Therefore, new technology looks to reduce this burden [45], but until these tools have been validated in athletes and dietary intake can be objectively and accurately tracked on a daily basis with ease, the transient nature of eating behaviours and dietary intake challenge the assumption that data collected during a short period of time are representative of days that were not assessed [22]. Furthermore, prospective tools such as this are limited by desirability bias [22].

Retrospectively, diet recalls and diet history assessments [22] can be used in a similar fashion to food logs and coded and analysed with software to determine dietary intake. While these tools have less athlete and practitioner burden than food logs, they are limited by recall error and the data acquired are not easily quantified with accuracy [22]. Practitioners may find it more useful to simply use these tools to qualify eating behaviours [22]. These retrospective tools are often used within the nutrition assessment (step 1) and nutrition monitoring (step 4) steps of the NCPM [21].

Often, practitioners will find it easier and more useful to quantify eating behaviours [22] rather than code and analyse data to quantify dietary intake. Tools such as Food Frequency Questionnaires [46] can quantify certain eating behaviours [10, 22], providing a quantitative alternative to coding and analysing data at a dietary intake level. Specifically, the Athlete Diet Index has been developed for and validated in athletes, with the purpose of assessing eating behaviours around training and for aspects of diet quality [43, 47]. Food Frequency Questionnaires and the Athlete Diet Index are retrospective tools and therefore prone to recall error due to their reliance on memory [22]. Fortunately, low-burden, prospective assessment tools can also be created for athletes to self-assess eating behaviours. These tools rely less on memory than retrospective tools, although often at the expense of external validity through potential increases in desirability bias [22]. Tools such as the Food Frequency Monitoring Tool (FFMT) are easier to implement on a daily basis than food logs or intake tracking software and allow for objective quantification of eating behaviours such as food choices and feeding frequency [48]. It may be warranted to periodize use of the FFMT throughout a year to minimize recording fatigue and maintain accuracy at key times. Additionally, assessment questions related to other eating behaviours (i.e. portion sizes and feeding time) can be developed and implemented on a daily basis with relative ease (e.g. what time did you eat breakfast?).

Athlete Self-Report Monitoring (ASRM) [49] can be used to subjectively assess dietary intake using a rating scale on a daily basis. This provides another efficient, prospective assessment tool option, although the focus on assessing dietary intake rather than eating behaviours makes ASRM more nuanced and subjective than the previously mentioned FFMT. Athletes require adequate understanding of nutrition requirements and nutrient content of the foods they consume to complete ASRM accurately and consistently. For athletes who have adequate nutrition knowledge, ASRM is a viable and practical option. Similar to the FFMT, it may be warranted to periodize the use of ASRM to minimize recording fatigue.

#### Tier 2: Development Tools

The NCPM nutrition intervention (step 3) step [21] was discussed earlier as the delivery of an action plan to address an identified capability, opportunity, or motivational issue, but action plans can also occur at an eating behaviour and dietary intake level. Prescribed plans can enable eating behaviours [34] and may come in the form of food plans that prescribe specific eating behaviours and nutrition plans that prescribe specific dietary intake. This approach can alter eating behaviours and dietary intake quickly; however, adherence to changes in eating behaviours and dietary intake as a *direct* result of these plans is low [50, 51], compared to *indirectly* through increases in capability, opportunity, or motivation. Non-compliance with food and nutrition plans is also common, even when there is intention to follow the plan [52]. Compliance with specific dietary intake may not even be possible without adequate underlying capability, whereas recommendations for general eating behaviours may be easier to implement, albeit less specific, as they do not rely on the athlete’s ability to code food into nutrients. Alternatively, some athletes may find it easier to adhere to a nutrition plan over a food plan given the relative increase in opportunity, as there are many different ways to achieve a specific dietary intake.

Food and supplement provision are also common sport nutrition services utilized to improve eating behaviours and dietary intake [34]. Food and supplement provision reduces the need for an athlete to be capable of making appropriate eating behaviour decisions. It may also address opportunity barriers such as decreasing financial requirements or time constraints, without requiring changes to an athlete’s awareness of the problem. For less popular behaviours, Nudge Theory [53] suggests food and supplement provision may modify ‘choice architecture’ enough to create behaviour change, potentially reducing motivational barriers.

### Tier 3: Consequences of Dietary Intake

The impact of appropriate dietary intake on the performance of athletes is well established [1]. Dietary intake is a critical component of performance and health indicators, including but not limited to: optimizing body composition; maintaining adequate energy availability; improving biochemical indicators; maximizing recovery from training and/or adaptation to training; mediating sleep quality and quantity; supporting the immune system; and improving performance readiness [1, 54, 55]. Dietary intake can also include the use of supplements such as creatine, beta-alanine, sodium bicarbonate, and caffeine as ergogenic aids to improve performance [1]. This tier revolves around measuring adaptations to training and readiness to perform and the feedback process used to shape eating behaviours, dietary intake, and their determinants.

#### Four Pillars of Athlete Development

Athlete development models categorize development into four pillars (technical skills; tactical skills; physiological skills (sometimes termed physical skills); and psychological skills (sometimes termed social, mental, and/or life skills)), after moving beyond stages designed to build physical literacy [56]. Appropriate dietary intake facilitates development within these pillars by augmenting adaptations to training and/or enhancing readiness to perform. Once the ability to sustain and modify eating behaviours has been established, and consistent eating behaviours have been demonstrated, the goal of sport nutrition services shifts to optimizing these adaptations and improving readiness to perform at critical periods through appropriate dietary intake. Identifying the specific desired adaptations and readiness requirements within the pillars of development should be driven by sport experts and will vary between and within sports and individual athletes. Physiological examples include improving body composition and energy availability and increasing glycogen storage. A psychological example could be improving the management of disordered eating. Technical and tactical examples include improving skills acquisition and decision-making, respectively. Once desired adaptation requirements and readiness requirements are identified, appropriate dietary intake can be informed by existing literature and expertise, to an extent. However, given the transient and uncertain nature of eating behaviours and resulting dietary intake, assessing key downstream variables helps identify whether desired adaptations and readiness to perform are adequately being achieved and provides an opportunity to adjust and/or reinforce the use of upstream development tools.

#### Tier 3: Assessment Tools

Assessing adaptation to training stimulus and determining readiness to perform is a standard practice in high-performance sport settings. Relevant to nutrition services, common areas for assessment include physique, hematological, clinical–physical, clinical–psychological, hydration, and energy requirements. The same type of Tier 1 psychological assessment tools can be used at this tier given the bidirectional relationship that eating behaviours and dietary intake can have with psychological factors, such as in the case of low energy availability [28, 29]. It is important to note that this is not an exhaustive list of the available assessment tools, or a description of how to use these tools. This list focuses on the more ‘common’ tools used by sport nutritionists, as outlining a complete list of functional assessment and performance analysis tools is beyond the scope of this paper. Table 2 contains assessment tools for assessing consequences associated with dietary intake.

**Table 2:** Overview of common assessment tools for consequences of dietary intake, including markers being observed, measurements taken, and tools used

Type	Markers	Measures	Tools
Physique	Mass Stature Fat mass Fat free mass Muscle mass Bone mass Bone density	Weight Height Girths Breadths Skinfolds Density	Scale Stadiometer Caliper Measuring tape Air displacement plethysmography Bioimpedance analysis/spectroscopy Dual energy X-ray absorptiometry
Hematological	Nutrient availability Nutrient stores Physiological system function Metabolic output	Testosterone (free, total) Estradiol Progesterone Follicle-stimulating hormone Luteinizing hormone Thyroid stimulating hormone Triiodothyronine Thyroxine Leptin Blood lactate Blood glucose Complete blood count Ferritin Transferrin Iron-binding capacity Vitamin D Vitamin B12	Laboratory-based bloodwork Point-of-care microcollection
Clinical–physical	Malnutrition Energy deficiency	Affect Body language Wounds (Skin) Vital signs Blood pressure Heart rate Temperature Subjective score	Physical exam by expert Heart rate monitor Blood pressure monitor Low Energy Availability in Females Questionnaire
Clinical–psychological	Eating disorders Disordered eating	Subjective score	Eating Disorder Examination 17.0 Eating Disorder Examination Questionnaire 6.0 Athlete Milieu Direct Questionnaire Version 2 Brief Eating Disorder in Athletes Questionnaire Version 2 Eating Disorder Screen for Athletes
Hydration	Urine concentration Total body water	Urine colour Urine specific gravity Intracellular fluid Extracellular fluid	Armstrong urine chart Urine refractometer Bioimpedance analysis/spectroscopy
Energy requirements	Basal metabolic rate Exercise energy expenditure Activities of daily living Total daily energy expenditure Fuel usage Energy availability	Resting metabolic rate Heart rate Metabolic equivalents Respiratory quotients	Metabolic cart Heart rate monitor Accelerometer Predictive equations

#### Tier 3: Development Tools

Given that Tier 3 is downstream of the actions of eating, there is no direct development that can occur. Indirectly, information gathered using Tier 3 assessment tools can be used (intentionally or unintentionally) to inform upstream decision-making. Therefore, using feedback from Tier 3 assessment tools to reinforce and/or adjust eating behaviours and dietary intake can be the primary development tool used to shape athletes at this tier. The appropriate selection of key downstream variables for assessing adaptation and readiness is important to inform the use of and response to upstream development tools.

## Applications of the Approach

Long-Term Athlete Development frameworks already exist, with many aiming to develop athletes from a foundation of physical literacy through to optimal competition performance [56]. These frameworks can serve as aides for sport coaches, strength and conditioning coaches, therapists, and performance analysts whose roles involve the development of physical traits in training environments. However, there is a dearth of resources to aid practitioners such as sport nutritionists and mental performance consultants in the development of the ‘lifestyle’ components that take place in the home environment without supervision. Additionally, certain populations and environments have unique dietary intake requirements or challenges that need to be considered when developing athletes [1]. Similarly, short-term athlete development may be required in situations where shifts in dietary intake requirements occur, such as return-to-play from injuries and energy deficiencies; athlete crossover between sports; shifts in physique and/or physique requirements; travel and competition environments; and variations in training goals through volume training, strength/power training, altitude training, and heat acclimation training [1]. To meet these unique dietary intake requirements, unique eating behaviours must be demonstrated, but there is a lack of resources to guide practitioners through this athlete development process. This approach has been presented as a starting point to guide practitioners and should be considered until future work allows for refinement and validation.

This conceptual nutrition approach provides three tiers to nutrition development and assessment, with each tier providing a foundation for the next. Moving through tiers can be a linear or iterative process, and this approach can be used to guide decision-making at a group or individual level. At a group level, servicing for junior athletes can focus on Tier 1 assessment and development tools, with ‘next generation’ groups receiving servicing using Tier 2 assessment and development tools, and reserving Tier 3 assessment and development tools for elite, senior athletes. Additionally, athletes can be grouped based on unique dietary intake requirements, to allow for development to be more specific to their needs at each tier, and assessment tools within the tier and downstream can be used to gather data and perform gap analysis to determine development requirements within the group.

At an individual level, with assessment of key factors at each tier, information can be used to guide development within that tier, or fed back upstream to determine development needs. The NCPM terms this step as nutrition diagnosis (step 2), the process of identifying and labelling nutrition problems prior to implementing an intervention [21]. As an athlete develops, less time should be spent in upstream tiers and more time can be dedicated to downstream tiers. Similarly, as an athlete develops, more emphasis should be placed on specialized assessment and individualized feedback to reinforce and/or adjust upstream factors, while at early stages, focus can be placed on general development and assessment of the determinants of eating behaviours. New services at subsequent tiers can be added as development occurs, with or without the removal of prior services. In ideal situations, athletes operating at a Tier 3 level will have the ability to quickly and sustainably make appropriate adjustments whenever data suggest a change is needed; however, in many cases, gaps in ability will be identified throughout the entire process, and development will need to continue at all three tiers.

It may be practical to deliver services at a group level early in development, with needs being more general. As athletes reach elite levels, individualized support is likely warranted, decreasing the ability and benefit of servicing at a group level. In any case, it is vital that development begins at Tier 1 given the contrast between the sustainability and the rate of development that will occur. With the lack of direct correlation with health and performance, the direct benefits of addressing determinants (e.g. increasing nutrition knowledge) may be delayed and therefore should be developed before optimizing immediate performance is paramount. Fortunately, determinants such as nutrition knowledge are durable qualities and should largely remain once instilled within an athlete, allowing for a shift in the focus of service delivery when in proximity to major competition, key periods of growth and maturation, important blocks of training, when unforeseen health concerns arise, or the ‘prime’ of a career. In contrast, eating behaviours and dietary intake are transient and can change daily, but are more directly related to desirable adaptations and readiness to perform when needed, allowing for this approach to be compatible within existing dietary intake recommendations for special populations (e.g. youth athletes) and long-term development frameworks.

## Conclusion

Cultivating appropriate eating behaviours to ultimately enhance health and performance is a complex and interrelated problem, requiring a holistic solution. Therefore, an approach was designed to aid practitioners in nutrition-related servicing decisions. This approach utilizes three tiers: Tier 1 focuses on the upstream determinants of eating behaviours; Tier 2 focuses directly on eating behaviours such as food choices, portion sizes, feeding frequency, and feeding time and dietary intake such as caloric intake and timing, nutrient intake and timing, fluid intake and timing, and supplement intake and timing; and Tier 3 focuses on the downstream consequences of dietary intake across four pillars of development.

## Data Availability

Not applicable.

## Abbreviations

DONE: Determinants of Nutrition and Eating
COM-B: Capability, Opportunity, Motivation-Behaviour
RD: Registered Dietitian
NCPM: Nutrition Care Process and Model
FFMT: Food Frequency Monitoring Tool
ASRM: Athlete Self-Report Monitoring
