# Correction: A Proposed Conceptual Sport Nutrition Approach for Athlete Development and Assessment: The Athlete Nutrition Development Approach

**DOI:** 10.1186/s40798-023-00561-z

**Published:** 2023-02-18

**Authors:** Kevin Iwasa-Madge, Erik Sesbreno

**Affiliations:** 1 Canadian Sport Institute Ontario, Toronto, ON Canada; 2 Institut National du Sport du Quebec, Montreal, QC Canada; 3 McGill University, Montreal, QC Canada; 4 French-Speaking Research Network for Athlete Health Protection and Performance (ReFORM), Montreal, QC Canada

**Correction : Sports Medicine - Open (2022) 8:142** **https://doi.org/10.1186/s40798-022-00532-w**

The original article [1] contained an error in the spelling of co-author, Erik Sesbreno’s name which has since been amended.

